# Implementing Key Strategies for Successful Network Integration in the Quebec Substance-Use Disorders Programme

**DOI:** 10.5334/ijic.2457

**Published:** 2016-04-05

**Authors:** Marie-Josée Fleury, Michel Perreault, Guy Grenier, Armelle Imboua, Serge Brochu

**Affiliations:** Department of Psychiatry, McGill University, Montreal, QC, Canada; Douglas Hospital Research Centre, Verdun, QC, Canada; Douglas Hospital Research Centre, Montreal, QC, Canada; Department of Criminology, Université de Montréal, Montreal, QC, Canada

**Keywords:** substance-use disorders, integration, services, network

## Abstract

**Background:**

Fragmentation and lack of coordination often occur among organisations offering treatment for individuals with substance-use disorders. Better integration from a system perspective within a network of organisations offering substance-use disorder services can be developed using various integration strategies at the administrative and clinical levels.

This study aims to identify integration strategies implemented in Quebec substance-use disorder networks and to assess their strengths and limitations.

**Methods:**

A total of 105 stakeholders representing two regions and four local substance-use disorder networks participated in focus groups or individual interviews. Thematic qualitative and descriptive quantitative analyses were conducted.

**Results:**

Six types of service integration strategies have been implemented to varying degrees in substance-use disorder networks. They are: 1) coordination activities-governance, 2) primary-care consolidation models, 3) information and monitoring management tools, 4) service coordination strategies, 5) clinical evaluation tools and 6) training activities.

**Conclusion:**

Important investments have been made in Quebec for the training and assessment of individuals with substance-use disorders, particularly in terms of support for emergency room liaison teams and the introduction of standardised clinical evaluation tools. However, the development of integration strategies was insufficient to ensure the implementation of successful networks. Planning, consolidation of primary care for substance-use disorders and systematic implementation of various clinical and administrative integration strategies are needed in order to ensure a better continuum of care for individuals with substance-use disorders.

## Introduction

According to the National Epidemiologic Survey on Alcohol and Related Conditions, the 12-month prevalence rates for alcohol abuse and dependence in the United States for 2001–2002 were 4.7% and 3.8% [1], respectively, while 12-month prevalence rates for drug abuse and dependence were 1.4% and 0.6% [2]. In Canada, 4.4% of the population 15 years and older had a substance-use disorder (abuse or dependence) in 2012, with alcohol dependence most prevalent at 3.2% [3]. In Quebec, 12-month prevalence rates in 2002 were 2.2% for alcohol and 1.1% for drugs [4].

Substance-use disorders are often associated with other problems such as high risk for early mortality, infectious or chronic physical diseases, poverty, domestic violence, incarceration and homelessness [5]. Moreover, multi-drug use [6,7] as well co-occurring substance-use disorders and mental health disorders [8] tend to be the rule, resulting in poor outcomes such as the exacerbation of psychiatric symptoms, poor functioning and prognosis, and high risk of relapse [9,10].

According to population surveys, individuals with substance-use disorders only are reluctant to use healthcare services [11,12], whereas those with co-occurring disorders are usually high users [13]. Some studies have found a high prevalence of healthcare service use among users of substance-abuse treatment centres [14,15]. For example, an Australian study reported that 60% of 615 heroin users had consulted a general practitioner in the previous month in connection with their substance-use disorders [16].

Individuals with substance-use disorders faced with multiple needs often find it necessary to consult a number of professionals and resources, including primary care as well as specialised services [17]. However, substance-use disorders are not always detected by healthcare professionals in regular healthcare services, just as mental or physical disorders may be overlooked in substance-use disorder services [18]. Furthermore, substance-use disorder services are often characterised by fragmentation and lack of service coordination [19]. In most countries, services for substance-use disorders and mental health disorders are provided in silo or sequentially rather than being integrated, which undermines optimal care for patients [20].

Overall, integrated treatment has proven more effective than standard treatment for co-occurring substance-use disorders and severe mental health disorders (e.g. schizophrenia, bipolar disorder) [21,22,23,24]. Yet research on the effectiveness of integrated treatment for co-occurring substance-use disorders and common mental health disorders (e.g. anxiety and depression) [25,26] or substance-use disorders and personality disorders [27] is less conclusive. As well, a great number of individuals affected solely by substance-use disorders or by mental health disorders also need some degree of coordinated care. Studies have found that service integration from a systems perspective may improve access to services for individuals with substance-use disorders and increase positive outcomes, including reduction of substance-use levels [28], improvement in mental and physical health [29], decrease in hospitalisation rates, inpatient days and emergency services use [30], as well as overall healthcare savings [31].

Better integration among organisations offering substance-use disorder services can be developed systemically by implementing various integration strategies throughout the network at the administrative (managers and coordinators) and clinical (staff) levels [32]. A ‘network’ may be defined as a set of organisations distributing a continuum of coordinated services to a defined population [33]. Of the few available studies on the overall integration of healthcare systems (macro-level analysis), we did not find any related to substance-use disorder service networks, as compared with the relatively high number of articles targeting integrated substance-use disorder treatment from the client perspective (micro-level analysis). In this context, the Quebec system provides an interesting case as Quebec has established a health and social services system over the past decade that includes a substance-use disorder programme operating within local service networks as opposed to within traditional institutions charged with a specific mission in substance-use disorders. This study aims to identify integration strategies implemented in Quebec substance-use disorder networks and to assess their strengths and limitations. We hypothesise that successful network integration requires the adequate implementation of a large number of integration strategies at both the administrative and clinical levels.

## Study context

### The Quebec Substance-Use Disorders Programme

Table 1 presents Quebec organisational and individual providers of substance-use disorder services. The main substance-use disorder providers are public-sector substance-abuse treatment centres, offering specialised services in the regional networks (*n* = 16; one per region). There are also 91 certified private or community-based substance-abuse treatment centres that offer treatments for individuals with substance-use disorders, mostly intensive inpatient rehabilitation. Eighty percent of the substance-use disorder service programme budget is allocated to public substance-abuse treatment centres, versus 15% for certified private or community-based substance-abuse treatment centres, and 5% for other services, which are mainly primary-care services [34].

**Table 1:** Quebec substance-use disorder providers.

Type	Services	Number

Public substance-abuse treatment centres	Specialised services	16
Certified substance-abuse treatment centres^1^	Specialised services	91
University hospital centres	Specialised and ultra-specialised services	3
Health and social service centres	Primary care	93
Psychologists/psychotherapists who treat alcohol-use disorders^1^	Primary care	213
Psychologists/psychotherapists who treat drug-use disorders^1^	Primary care	301
General practitioners who follow up individuals with substance-use disorders	Primary care	Unknown
Alcohol anonymous (AA) groups	Primary care	1385
Narcotics anonymous (NA) groups	Primary care	264
Cocaine anonymous (CA) groups	Primary care	31

^1^These are private or community-based organisations often supported by financial contributions from clients who fully or partially defray the costs of services received. With the exception of the AA, NA and CA, all other organisations are public, with no charge for services.

### Quebec substance-use disorder regulation

In Quebec, health and social services, including mental health and substance-use disorder services, are divided into service programmes (*n* = 9) and managed at three levels of regulation: provincial, regional and local. The Quebec Ministry of Health and Social Services assumes general governance and control over provincial health care [35]. Unlike other service programmes (e.g. for mental health disorders), substance-use disorder services are not integrated into the health sector, but into social services. The current ministerial strategic plan for the substance-use disorder programme [36] defines the roles of public substance-use disorder providers as follows: Regional health agencies (*n* = 16) are responsible for planning, organising, coordinating, budgeting and evaluating health and social services in their respective regions. The role of local community service centres, or primary-care services, involves substance-use disorder detection, provision of early treatment or psychosocial intervention, medical follow-up and referral of clients at risk, or those stabilised after treatment, to specialised services. Local networks (*n* = 93) were created in 2005 in the context of a major healthcare reform; each local community service centre was then merged with acute care hospitals and nursing homes, forming a health and social service centre (*n* = 93). The latter, and specifically the local community service centres, were mandated to coordinate all services in their respective local jurisdictions in order to better respond to the needs of their populations and to improve service integration and the quality of care. The health and social service centres/local community service centres had to develop strategic care planning for each healthcare service programme, including substance-use disorders, in conjunction with all local service providers. Thus, a local substance-use disorder service network is mainly composed of a health and social service centre/local community service centre, a public substance-abuse treatment centre and some certified substance-abuse treatment centres.

## Methods

### Design and study population

The study was conducted in two regions of Quebec (Canada): Montreal and Mauricie. Montreal is Quebec’s largest urban centre, while the Mauricie region includes both urban and rural areas. These two distinct regions were selected in order to describe the organisation and practices of substance-use disorder services in different contexts (urban and rural areas), and at different levels: (1) local service networks: with the focus on health and social service centres responsible for primary healthcare services and coordination with partners; (2) regional networks: with the focus on public substance-abuse treatment centres, the main specialised substance-use disorder providers responsible for coordination between primary-care and specialised services at the regional level, and with related partners such as university hospital centres.

Table 2 lists the main network integration strategies reported in the literature, which served as the analytical framework for the study. Administrative integration strategies include information systems and inter-organisational agreements (e.g. service contracts). Clinical integration strategies include individual service plans, training activities, clinical evaluation tools and the like.

**Table 2:** Integration strategies* – analytical framework.

**A. Administrative**			

1. Governance system:	▪ Steering committees at strategic, tactical and operational levels		
2. Management system for the network:	▪ Policies, orientations and planning	▪ Care trajectory	▪ Primary-care models
	▪ One-stop service	▪ Inter-organisational agreements/service contracts	▪ Information and monitoring management tools
**B. Clinical**			
1. Service plans	▪ Intervention plans	▪ Individual service plans	▪ Treatment/follow-up protocols
2. Case management	▪ Case managers	▪ Intensive case management ▪ Assertive or non-assertive community treatment	
3. Referral mechanisms between organisations			
4. Joint programmes			
5. Training, clinical coaching and inter-organisational internships			
6. Integrated care			
7. Shared staff between organisations			
8. Other clinical tools	▪ Needs assessment tools, uniform registration forms, report cards, consent forms, etc.		

*Adapted from: Ref. [32].

### Data collection

Data sources included mainly qualitative interviews. Relevant documents from organisations, networks and the substance-use disorder literature were also consulted. Sociodemographic data were collected from all participants using a brief questionnaire. A semi-structured interview guide based on a literature review and consensus among researchers was developed and adapted to each organisation and participant groups. Focus groups of five participants each, on average, were preferred as an effective way of collecting information from multiple participants simultaneously, while promoting group dynamics [37]. Individual interviews were conducted in organisations where only one participant had been recruited to the study. In total, 5 semi-structured interviews (average duration: 1 hour) and 19 focus groups (average duration: l.5 hours) were conducted between January and November 2013. The following dimensions were covered by the interview guide: (1) organisational characteristics (e.g. vision, roles and responsibilities, coordination of services); (2) service integration and organisation within local or regional networks (e.g. level of integration, coordination strategies implemented); 3) processes used to detect individuals with substance-use disorders or alcohol and drug consumption problems, referral mechanisms to specialised substance-use disorder services and outcomes; (4) factors that facilitate or hinder substance-use disorder service organisation and coordination and (5) recommendations for improving substance-use disorder service networks. A single question was asked in relation to each dimension (e.g. ‘In your opinion, how, and to what extent, has this dimension been integrated within your organization or network?’), followed by sub-questions eliciting additional information. The focus groups and interviews were audio-recorded and transcribed, and each participant was identified by a number. All participants signed a consent form. The multi-site study protocol was approved by the Ethics Board of the Montreal public substance-abuse treatment centre.

### Analysis

Quantitative descriptive analyses were used to describe participant sociodemographic characteristics. For the qualitative data, a six-step thematic analysis was conducted [38] including: (1) transcription of interviews; (2) preliminary reading of transcriptions; (3) selection and definition of the units of meaning; (4) development of an analytical framework; (5) separation of content into meaningful units of information and (6) coding using the analysis grid. The analytical framework and the coding structure were based on the interview topics and took into consideration integration strategies identified in Table 1, while allowing for the inclusion of emerging themes. They also took into account participant types, organisations, regional and local networks. A research report was produced for the two participating regions summarising the main results, which were then reviewed and discussed by all researchers, serving as the basis for the present article.

## Results

### Characteristics of study participants

There was a total of 105 participants in the study, including 65 clinicians (social workers, nurses, psycho-educators) and 40 managers (directors, programme managers, coordinators) (Table 3). They were involved in providing services to individuals with substance-use disorders, in both local and regional substance-use disorder networks. They represented the following 12 organisations: the substance-use disorder and homelessness service programmes of the Health and Social Services Ministry, two regional health agencies, four local community service centres, one university hospital centre, two hospital centres affiliated with two of the selected health and social service centres and two public substance-abuse treatment centres. A key respondent was identified within each organisation and asked to assist with recruitment of clinicians and managers in their respective organisations. In all, 39 clinical or management teams were met (22 in Montreal, 16 in Mauricie and 1 from the Health and Social Services Ministry). The breakdown of teams was as follows: 11 in hospitals, 21 in local community service centres, 4 in public substance-abuse treatment centres, 2 in the Regional Health Agencies and 1 from the Health and Social Services Ministry.

**Table 3:** Characteristics of the participants (*N* = 105).

Variables		Mean% (SD)

Region	Montreal	61.2
	Mauricie	38.8
Sex	Male	28.6
	Female	71.4
	20–29	16.3
	30–39	37.5
Age (years)	40–49	26.0
	50–59	15.4
	≥60	4.8
Education	High school	5.8
	University	94.2
Field of study	Social sciences	65.0
	Health sciences	32.0
	Other	2.9
Function	Clinicians	61.9
	Managers	38.1 (40)
		
Years of experience in the local network		15.66 (9.49)
Years of experience in the current organisation		9.96 (8.00)
Years of experience in the substance-use disorder programme		9.91 (9.26)

### Network characteristics

Table 4 describes six service integration strategies implemented in the two regions and four local networks studied. They were identified as: (1) coordination activities-governance; (2) primary-care consolidation models; (3) information and monitoring management tools; (4) service coordination strategies; (5) clinical evaluation tools and (6) training activities. Table 4 also indicates the level of implementation associated with each of the strategies at the regional or local level. Examples of emblematic quotations from the interviews are illustrated in Box 1, followed by a description of service integration strategies and the factors hindering or facilitating their optimal integration.

**Table 4:** Summary of service integration strategies implemented.

Strategies (type)	Description	Level of implementation

Governance (*administrative*)		
Steering committee	Initiative of the Regional Health Agencies to oversee organisational development of substance-use disorder services.	Implemented in one region
	Initiative of health and social service centre coordinators to oversee the development of substance-use disorder services at the local level.	Implemented in one region
Planning	Local action plan or clinical project related to substance-use disorders that identify: (1) objectives to be achieved; (2) organisations (or people) responsible and collaborators; (3) expected results or indicators of success and measures required; (4) deadlines [36].	Implemented in two local networks
Primary-care models in health and social service centres (*administrative*)		
	Primary-care consolidation models that aim to provide substance-use disorder services in parallel with other health service programmes offered at health and social service centres. All models enhance services with added substance-use disorder expertise, given the lack of knowledge on substance-use disorders among primarycare professionals.	Moderately implemented at the local level
Information and monitoring management tools (*administrative*)		
‘I-CLSC’: information system on consumers and services in local community service centres. ‘SIC-SRD’: information system for substance-use disorder rehabilitation services in public substance-abuse treatment centres.	Systems set up to support clinicians and managers in better understanding the substance-use disorder clientele, to improve quality and efficiency of services provided in their respective organisations and to provide information on healthcare governance (to Ministry of Health and Social Services and regional agencies) with emphasis on data monitoring and resource control.	Moderately implemented in each local network
Coordination strategies (*administrative or clinical*)		
Care trajectory	Administrative strategy established by health and social service centres to facilitate understanding by organisations of their role regarding integration of the substance-use disorder programme and also understanding by clinicians of their responsibilities for identification, screening and follow-up of clients with substance-use disorders.	Implemented in two local networks
Service contracts	Administrative strategy used in health and social service centres and substance-abuse treatment centres as one of the formalised mechanisms available for soliciting their partners and gaining their active support [36]. Service contracts facilitated access to and continuity of services.	Not sufficiently implemented in each local network
Service contracts	Administrative strategy used in health and social service centres and substance-abuse treatment centres as one of the formalised mechanisms available for soliciting their partners and gaining their active support [36]. Service contracts facilitated access to and continuity of services.	Not sufficiently implemented in each local network
Emergency room liaison teams	Clinical strategy that tracked individuals with substanceuse disorders in emergency rooms and directed them to the appropriate services. Emergency room liaison teams consist of clinicians from substance-abuse treatment centres working in partnership with ER clinical teams and hospital units. They provide substance-use disorder clients with quick access to substance-abuse treatment centres or other necessary services.	Implemented in the two regions
Joint programme or co-location	Clinical strategy to establish shared services across more than one organisation to ensure coverage of the required range of services. Joint programmes involve the sharing of staff; co-location involves the sharing of services.	Not sufficiently implemented in each local network
Individual service plans	A clinical strategy based on mutual agreement among several service providers, the client or his/her representative and members of his/her entourage that defines client care or service objectives [39]. Individual service plans are designed to ensure a better connection among the services used by clients, to reduce duplication, to better match services to client needs, to ensure effective service continuity and to allow clients to actively participate in decisions concerning them.	Not sufficiently implemented in each local network
Case management	Case management is a method of ensuring accessibility and continuity of care for clients with mental health disorders according their specific needs [40].	Not implemented into services for individuals with substance-use disorders only
Assertive community treatment	Strategy based on the collective responsibility of a team (e.g. psychiatrist, nurses, social workers) who provide intensive treatment services, rehabilitation and monitoring in the living environment of individuals with serious mental health disorders and related functional disability, as well as very high risk of multiple admissions (‘revolving door’ syndrome) [41].	Not implemented into services for individuals with substance-use disorders only
Intensive case management	An intervention by case managers that ensures continuity of care for individuals with mental health disorders who are more apt to integrate into the community than clients receiving Assertive Community Treatment [42].	Not implemented into services for individuals with substance-use disorders only
Evaluation/clinical tools (*Clinical*)		
substance-use disorder Screening questions in LSCCs	3–6 questions.	Moderately implemented in each local service network
Assessment of Needs for Help for Alcohol, Drugs or Gambling in local community service centres (DÉBA: *Dépistage/évaluation du besoin d’aide*) [43]	Standardised tools designed to help direct clients to the service(s) or institution(s) best suited to their needs. Contents: alcohol: 28 questions; drugs: 24 questions; gambling: 8 questions. [36].	Not sufficiently implemented in each local service network
Assessment of Needs: NIDEM (*Niveau de désintoxication: évaluation par les intervenants médicaux*), NIDEP (*Niveau de désintoxication: évaluation par les intervenants psychosociaux*). In public substance-abuse treatment centres: ASI (Addiction Severity Index) Global Appraisal of Individual Needs	Standardised evaluation tools used in emergency rooms: NIDEM assesses level of detoxification and is used by medical workers; NIDEP: assesses level of detoxification and is used by psychosocial workers; ASI [44]. Seven scales: Drugs, Alcohol, Medical condition, Family and social relationships, Psychological condition, Work and resources, Legal situation. Global Appraisal of Individual Needs [45]: A specialized evaluation tool to assess, plan treatment and monitor results. Eight sections: Background, Substance use, Physical health, Risk behaviours and disease prevention, Mental and emotional health, Environmental and living situation, Legal situation, Vocational situation.	Adequately implemented in each local service network
Training activities (*Clinical*)		
Cross-training	A strategy to enhance collaborative environments by simultaneously training clinicians with expertise in substance-use disorders or mental health disorders. Interagency exchange days for case discussions, especially for dual diagnosis, were recommended as a strategy by the *Centre of Substance Abuse Treatment* in United States and Health Canada to improve the effectiveness of interventions and the development of an integrated system of care for this clientele [46,47].	Moderately implemented at the regional and local levels

**Box 1. Emblematic quotations**

**Coordination Activities-Governance**

*Local Plan:* Next year, we’ll be investing in substance-use disorder teams, and creating an action plan along with the team leaders to do something that also fits with their reality and needs. As well, we’ll do this gradually, because we should not rush things. So, for example, we’ll start by offering a clinical consultation on these teams. And slowly we’ll introduce tools. (Manager 1, health and social service centre, Local Service Network 1, Montreal)

*Steering Committee:* In each territory, there’s a dependence ‘consulting body’, which reports to the health and social service centre. The health and social service centre hosts events, and brings together partners who are concerned by the issue. These partners include community organizations, education communities, law enforcement agencies, depending on the priorities that will have been identified in each territory. (Manager 2, Regional Health Agency, Montreal)

**Primary-Care Models**

*Model 1:* The challenge for substance-use disorder programs is mainly how to implement the program within and across the different institutions that don’t have teams specifically dedicated to substance-use disorder work. So the role of the specialist clinical consultant in substance-use disorders is to sensitize service providers working within each team on how to detect and track substance-use disorder problems. This involves training primary-care workers to take on these clients, to make them aware of resources available outside of the CSSS as a point of reference, and to work toward motivating those clients in our services who present with substance-use disorder problems. In terms of access to training, the consultant takes care of all programming, training plans, and dissemination of information to team members. (Manager 13, local community service centre, Local Service Network 2, Montreal)

*Model 2:* There is a kind of openness to detection among service providers when there is someone to whom clients may be referred – someone capable of taking the lead and supporting them. It’s a bit the same perspective we took when we created teams of expert trainers in the regions, which was to create a hard-core support around substance-use disorder workers in both primary care and specialist services, so that others could rely on them and feel reassured. (Manager 1, Ministry of Health and Social Services)

*Model 3:* Each of our CSSS has a dedicated substance-use disorder worker, whose job is to deal with clients. This worker is associated with the adult mental health team but can also help with the children’s team in the organization. That’s for primary care. (Manager 20, Regional Health Agency, Mauricie)

**Information and Monitoring Management Tools**

The data provided at the regional level are not very detailed, nor was there much information on the substance-use disorder population at the provincial level. The only way I was able to obtain a portrait of the substance-use disorder clientele for our territory was through annual reports of the substance-abuse treatment centre. This is a kind of roundabout way of finding out about the pool of clients we have, because if you look in our client information systems, things are poorly documented, and maintaining records on interventions related to substance-use disorders is a problem because this usually isn’t a priority for workers. (Manager 5, health and social service centre, Local Service Network 1, Montreal)

**Services Coordination Strategies**

*Emergency Room Liaison Team:* When we started having liaison teams in hospital emergency rooms, there was more openness from ER staff to identifying substance-use disorder cases because there was someone to whom we could refer them. There is someone who can provide support, who can take over. (Manager 1, Health and Social Services Ministry)

*Service Contracts:* We had a service agreement, but were unable to apply it, because there was always someone saying ‘no’ at the other end of the line, even if the agreement stipulated that it had to be done. So as one can see, formal agreements do not guarantee harmonious relationships. (Clinician 7, University Hospital Centre, Montreal)

*Joint Program:* The homelessness team in our local service network consists of two network institutions: the local community service centre and emergency residential services that are part of community organizations. So this means four workers for two different organizations who make up the homelessness team. The workers on the team share their expertise with clients living in the community. They bring their experience from each of their organizations and have developed a complementary approach that benefits everyone. This is unique in Quebec; it is a model that doesn’t exist elsewhere. (Clinician 43, local community service centre, Local Service Network 1; Mauricie)

*Co-location:* I find that there was good cooperation between the primary-care workers and those from the specialized substance-use disorder services whose offices are in close proximity. They are able to exchange ideas, discuss cases, and share their expertise and knowledge. (Clinician 47, health and social service centre, Local Service Network 2, Mauricie).

*Individual Service Plans:* Things are improving here, which is not usually the case province-wide. Each institution has to train coaches for implementing the Individual Service Plans; so we are looking to set this up. (Clinician 51, Public substance-abuse treatment centre, Mauricie)

**Evaluation Clinical Tools**

*DÉBA* (*Assessment of Need for Help for Alcohol*, *Drugs or Gambling in local community service centres*): These tools are somewhat daunting for some, maybe because of the more formal aspect of the questionnaire, although that is actually an advantage from my perspective. And then, when are we going to administer them? For example, when someone makes a request for services, not for substance-use disorder services but for related problems, or other problems? And then you realize after pre-registration that there is a problem with substance-use disorder, but someone has already done an hour-long evaluation, or an hour and a half on a bunch of issues, and this means doing another 45 minutes just for the DEBA. It's difficult to integrate the latter into a service organization, given the time that people have available. (Clinician 19. Local service network 1, Montreal)

**Training Activities**

*Basic Training*: Good progress has been made, precisely among the primary care workers. We had a first session that trained them on assessment tools. The second session was on the motivational approach. All health and social service centres were trained for that. (Manager 3, Regional Health Agency, Montreal)

*Cross-training*: Just to give an example, we gave training over 4 years … We had to train 300 workers over the entire region that we covered. At the same time, what we tried to do was a cross-training, a training of the network. This allowed us to create links with people, to work and integrate better. I think that these are the promising avenues at an intervention level for dual disorders. It allows for staff exchanges among organizations, so that they can get better acquainted with the types of clients referred to these services for substance-use disorders, and perhaps to have better access to data on service use as well. (Clinician 39, health and social service centre, Local Service Network, 1, Mauricie)

**Factors That Hinder or Facilitate Optimal Service Integration**

*Lack of Resources*: We don't have the resources to put in place (…) the other health and social service centres in our region (urban territories) have three full-time people for substance-use disorders. They have the time to start projects. They have the time for many things. The realities of the territory, having many workers to develop projects for a small clientele versus the Local Service Network 1, Mauricie, which has four times the population as we do. (Clinician 44, local community service centre, Local service Network 2, Maurice)

*Leadership and Overall Lack of Integrated Care*: The regional agency did not provide the leadership to organize and assist with development of a regional vision on what should comprise primary care substance-use disorder services. They based themselves on the existing Ministerial frame of reference, but this wasn't a regionally conceived plan that would elaborate in more concrete terms what should be done in the health and social service centre, and what should be done in specialized services; how the two should work together. (Manager 11, health and social service centre, Local Service Network 2, Montreal)

*Lack of Communication*: It needs to be coherent, because these are clients who move all around the territory soliciting everywhere they go. There are laws that bind us to the confidentiality aspect. Then there are resources: some institutions are much more concerned and ticklish about confidentiality, in terms of the kind of information they give out. (Clinician 6, health and social service centre, Local Service Network 1, Montreal).

*Reluctance to Change Practices*: When you try to implement a new tool, it's seen as extra work, as something complicated. We see that there's resistance, certain partners have set up the assessment tools, identification tools, follow-ups, etc. Other partners have been far more difficult to engage or even non-existent. (Manager, Public substance-abuse treatment centre, Mauricie)

*Differences in Values and Practices*: There is little place for occupational therapists, or social workers in the client vision, and all that. But I find that they are a major addition in a more global vision of health; but it's more difficult to introduce this kind of vision in medical or hospital services; sometimes there isn't a place for them, because the discourse is more medical. (Clinician 15, University Hospital Centre, Montreal)

*Staff Turnover*: There is something else, which is staff turnover. We have people who work 1 week, 2 weeks, so they are not very aware; they don't know others to whom they can refer clients. I would say that we need to work on this. (Clinician 63, Public substance-abuse treatment centre, Local Service 1, Mauricie).

*Lack of Training and Prejudices Against Substance-use Disorder*: What I understand from workers is that they are not necessarily comfortable about intervening with substance-use disorder cases. It's as if they think they need to be experts in order to work in the substance-use disorder program. By contrast, when they are in the health and social service centre, in the local community service centre (primary care), we talk about substance-use disorders but the clientele they see are not, in principle, ‘addicted’. There are inappropriate consumption habits, perhaps occasional issues; it may be an emerging substance-use disorder, but in principle the clientele they see are the ‘yellow lights’, not the addicts (‘red lights’). Their intervention is, in my sense of things, the same as any other psychosocial intervention. Yet I also have the impression that they have a kind of prejudice or uneasiness, whether they aren't sufficiently equipped or whether, as some say, it's personal. They are embarrassed to address the matter. They don't know what ‘Pandora's box’ they are going to open; they aren't comfortable in themselves talking about substance-use disorder, the ‘red lights’. (Clinician 22, Local Service Network 2, Montreal)

*Clients*: I think there is a certain distrust of institutional rehabilitation services for substance-use disorders. From my understanding of clients, for example, they think that the interventions are going to be controlling of their consumption, with objectives that they won't necessarily be able to attain. We speak about harm reduction, but you know, I have the impression that, for clients, this often means a reduction in consumption. So given all the problems that they are living with, they don't necessarily feel able to reduce their consumption, to take on therapeutic work around their substance use, to get help for the harmful consequences of consumption. (Clinician 2, health and social service centre, Local Service Network 1, Montreal)

### Coordination activities-governance

Key policy orientation documents were launched at the provincial level, but very little investment made in consolidating and integrating services. At the regional level, steering committees including key organisations in the substance-use disorder programme were put in place. One of the two regional health agencies met every 2 months with the substance-use disorder coordinators at all health and social service centres and with all substance-use disorder stakeholders once a year, in order to identify the role of each organisation and to coordinate regional substance-use disorder services. In the other study region, the substance-use disorder coordinators at each health and social service centre met every 6 weeks to discuss the delivery of substance-use disorder services, bringing questions on directions and priorities to the regional health agency, including questions about service integration.

### Primary-care consolidation models

Three types of models were implemented. In the first model (identified in one local network), a substance-use disorder consultant was attached to clinical teams that were not specialised in substance-use disorders. This consultant did not see individuals with substance-use disorders, but held case discussions with professionals, providing them with clinical evaluation tools and training. In the second model (identified in two local networks), the substance-use disorder consultant identified caseworkers, who acted as ‘SUD resource clinicians’ and ‘SUD specialist promotion agents’ on their respective teams. Finally, in the third model (identified in a local rural network), substance-use disorder caseworkers were integrated into the mental health team.

### Information and system monitoring management tools

System monitoring was implemented at the provincial level by the Health and Social Services Ministry in order to follow client trajectories and monitor the quality and efficacy of the substance-use disorder programme. I-CLSC (information system on consumers and services in local community service centres) and SIC-SRD (information system for substance-use disorder rehabilitation services in public substance-abuse treatment centres) are the main substance-use disorder system management tools (see Table 4), which gather information on types of service requests and services used, frequency of service use, clinical notes and other health-related information. In 2013–2014, a total of 6820 individuals received primary-care services in Quebec health and social service centres for alcohol, drug or gambling problems, which represents an increase of 22% over 2012–2013. Also in 2013–2014, wait times for specialised substance-use disorder services were less than 15 days for 75% of the requests made within all Quebec public substance-abuse treatment centres.

### Service coordination strategies

The administrative strategies identified included care trajectories and service contracts. The health and social service centres in two local networks established, for them and their partners, a care trajectory for individuals with substance-use disorders. Unlike mental health disorders for whom the care trajectory from primary to specialised care was clear, and organised through a local one-stop service, individuals in the substance-use disorder system could access substance-use disorder services through both primary-care services (local community service centres) and specialised services (substance-abuse treatment centres). This was a source of confusion, as primary-care and specialised services were not clearly delineated and was partly explained by the paucity of resources allocated to substance-use disorder treatment in primary care. Furthermore, in some local networks, service contracts were signed by both the health and social service centre and public substance-abuse treatment centre in an effort to optimise service coordination between them. Yet, according to some managers in those organisations, service contracts did not guarantee real service integration, given the many bureaucratic obstacles to service coordination in place.

Several other service coordination strategies were identified, including the development of emergency room liaison teams, joint programmes, co-location, referral mechanisms, individual service plans and the implementation of integrated care at different levels. Emergency room liaison teams have been successfully implemented in the four local networks. At the provincial level, 27 emergency room liaison teams specialised in the delivery of substance-use disorder services were implemented in 28 hospital emergency departments throughout 10 regions, including the two participating regions in the present study.

Some joint programmes or co-location experiences were identified. In one local network, for example, a team composed of clinicians from the health and social service centre, the public substance-abuse treatment centre and two community-based organisations was formed to deal with homelessness. In another local network, a public substance-abuse treatment centre service point was established on the same site as the health and social service centre in order to facilitate inter-organisational collaboration.

Concerning referral mechanisms, few organised referral systems were in operation especially between primary-care and specialised services. Substance-use disorder clients usually had to call the referral service on their own initiative. As a result, the referring organisation did not know whether clients had followed through. Client follow-up was lacking, in fact, and there were problems with access to, or continuity of, services. According to some clinicians, referrals to ultra-specialised services, including those for co-occurring disorders, were also underdeveloped and very difficult to access.

Individual service plans were developed for complex or chronic substance-use disorder cases, but were not often used. Concerning integrated care for co-occurring disorders [48], the closest testing of this model was conducted in Montreal clinics offering ultra-specialised services and involved the coordination of services among a public substance-abuse treatment centre and a mental health university institute. There was also an inpatient unit for individuals with substance-use disorders and complex conditions in a university hospital centre, where some beds for mental health disorders were provided. Finally, case management, assertive community treatment, as well as intensive case management were located mainly within mental health disorder services and were not implemented into services for individuals with substance-use disorders only.

### Clinical evaluation tools

Screening for individuals with substance-use disorders in local community service centres was relatively formalised and usually done at the reception/evaluation/orientation service desk, using a number of screening questions included in the admissions questionnaire. However, clinical teams were unlikely to screen clients as part of the medical treatment process, as some clinicians found the standardised clinical evaluation tool [43] developed for local community service centres too complex or long to complete. By contrast, client evaluation in hospital emergency rooms was systematised using two standardised clinical evaluation tools (see Table 4). In public substance-abuse treatment centres, clients were assessed automatically for substance-use disorders at admission using the Addiction Severity Index. This tool is to be replaced in future by the Global Appraisal of Individual Needs, an instrument used in several countries and translated into several languages. The Global Appraisal of Individual Needs is currently being piloted in Quebec.

### Training activities

Cross-training activities were conducted in various organisations within the four networks in order to increase knowledge on substance-use disorders and on local resources for substance-use disorders. The majority of respondents viewed cross-training on co-occurring disorders as a facilitator to networking and cooperation among clinicians from health and social service centres, and the public and certified substance-abuse treatment centres who were involved with substance-use disorders and mental health disorders. Cross-training also facilitated the referral process between programmes, supporting client follow-up and the development of individualised service plans.

Moreover, the public substance-abuse treatment centres offered training and consultations to health and social service centres/local community service centres and certified substance-abuse treatment centres in the four local networks. For their part, health and social service centres provided similar activities to their local partners (schools, jails, etc.). Respondents found this exchange of expertise particularly useful. Clinicians in one local network reported that training on substance-use disorder screening provided to teachers and adult education staff had the effect of facilitating referrals to specialised services.

Finally, the Health and Social Services Ministry offered basic training activities covering identification, screening and early intervention for substance-use disorders, as well as motivational interviewing, which is a recognised best practice for substance-use disorders and co-occurring disorders [49]. The Ministry also provided training on ‘Alcochoix’ [50], a prevention programme offered in 76 of the 93 health and social service centres for individuals at elevated risk for substance-use disorders. This basic training was targeted specifically to primary-care providers (health and social service centres/local community service centres) in line with implementation of the Ministerial guidelines. However, clinicians criticised this training somewhat as too general.

### Factors that hindered or facilitated optimal service integration and participant recommendations

Several factors hindered the optimal integration of substance-use disorder services. The greatest barrier, according to all respondents, was lack of funding and resources. Of the nine programmes within the purview of the Ministry of Health and Social Services, the substance-use disorder programme received only 0.32% of the budget envelope, which is the lowest funding allocation for all programmes. Although underfunding affected all services, primary-care services were most severely affected (5% of substance-use disorder service programme budget) [34]. Furthermore, according to some participants, organisational budgets were renewed without either service planning at the regional level or assessment of community needs [51]. This resulted in the inability of some health and social service centres to provide full-time specialist substance-use disorder workers. Finally, the fee-for-service payment system used in Quebec to remunerate general practitioners did not favour the appropriate management of vulnerable clients, such as those with substance-use disorders.

The lack of regional leadership was another hindering factor for service integration, according to some managers in the study. In one Regional Health Agency, a dearth of leadership in terms of coordinating services between substance-use disorder organisations was reported. Each health and social service centre was left to initiate its own service coordination strategies.

Poor dissemination of information among organisations, programmes and lines of service was another key hindering factor, according to managers. Lack of communication between primary-care and specialised services affected client follow-up, in turn. Moreover, the creation of links between public, community and private organisations offering substance-use disorder services remained challenging. The study findings also reflected differences in values and practices among various organisations.

Other factors hindering optimal service integration concerned professionals themselves. According to managers, some professionals were reluctant to change their practices or were not sufficiently well trained to engage in collaborative practices. Several professionals demonstrated prejudiced views toward individuals with substance-use disorders, classifying them as difficult or dangerous. Staff turnover also emerged as a major barrier to service integration and to knowledge translation following upon the substance-use disorder training.

Client beliefs and behaviours also emerged as a hindering factor, according to clinicians who participated in the research, for example those who denied their diagnosis, and/or refused a referral to specialised services. Moreover, clients who enjoyed a good therapeutic alliance with their service providers (e.g. at the local community service centre) were sometimes reluctant to accept a transfer when required, especially transfers between primary and specialised care (e.g. the substance-abuse treatment centre).

Among the factors that facilitated optimal service integration was strong engagement by the Ministry of Health and Social Services in fostering service integration among organisations, ministries and service programmes (e.g. programmes for mental health disorders, youth programmes, public health programmes). This had the effect of facilitating more effective prevention and treatment of substance-use disorders.

Study participants made a number of recommendations aimed at further strengthening primary care, while enhancing service integration and inter-organisational collaboration within the Quebec substance-use disorder Programme. Participants suggested that prevention services in primary care should be reinforced and more simplified assessment tools developed for clinicians relatively unfamiliar with substance-use disorders. They also recommended the adaptation of training programmes to the particular circumstances and needs of local teams, such as their level of involvement with substance-use disorder clients. From their perspective, clients needed to be better motivated and supported in accessing help from specialised services. To this end, they recommended a more formal collaboration between primary and specialised services that would entail clear referral mechanisms and service contracts, as well as increased opportunities for professional exchange among clinicians representing various organisations. Finally, participants considered particular attention to the development of tools for managing client information and monitoring as a key element for evaluating performance in substance-use disorder services and programmes.

## Discussion

The organisation of the Quebec Substance-use Disorder Programme does not differ substantially from substance-use disorder programmes in other countries with respect to its positioning at the interface of the health and social service system, the existence of several organisational types within the public, private and community sectors, or in terms of the fragmentation of primary-care and specialised services, more generally [52,53,54]. Overall, the problem of fragmentation can be explained as a function of the complexity of integration processes and the existence of a range of factors that hinder service integration. These factors affect services to the client as well, from initial evaluation to post-treatment follow-up [53]. As in Australia [55] and Germany [56], the Quebec practice of administering and funding substance-use disorder and mental health disorder services separately provides few incentives for collaboration. By contrast, the United Kingdom and United States have both adopted a multi-agency approach to substance-use disorder and mental health disorder service delivery over the past few years, offering integrated substance-use disorder/mental health disorder care that includes all partners [57]. Strong support from the Quebec government and substance-use disorder service providers to reform the Substance-use Disorder Programme is a key condition for implementing change [58,59].

Underfunding of substance-use disorder programmes, and particularly at the primary-care level, seems fairly wide-spread throughout the world [19,60,61]. This can be explained by the fact that substance-use disorder care is less related to medical treatment than other health problems [52]. The entrenched stigmatisation of individuals with substance-use disorders [62,63] also explains why few resources are invested in programmes for this population. Some authors have highlighted the negative effect of scarce resources on service integration [64,65]. Yet the need to secure resources in a context of scarcity also encourages public, private and community-based organisations to work together towards this shared goal [32,65].

Aside from resource constraints, other reported factors hindering the optimal integration of Quebec substance-use disorder services coincide with findings from other countries. They include lack of leadership [66], high staff turnover [60], the interest and capacity of clinicians to identify and treat substance-use disorders [61,67], diverging values and practices among organisations and professionals [66], poor communication and coordination between general practitioners and specialised substance-use disorder services [13], lack of follow-up and continuity of care [53] and insufficient motivation among substance-use disorder clients to enter into treatment [13].

Overall, our study reveals that few integration strategies were implemented among professionals, programmes or organisations operating in local or regional networks. This result confirms our initial hypothesis that successful network integration requires adequate implementation of a large number of integration strategies at both administrative and clinical levels. Quebec policymakers have focused primarily on clinical strategies in an attempt to improve the Substance-use Disorder Programme. According to the findings, three strategies were implemented more adequately than others and provided effective results in terms of integration and continuity of care. They included hospital emergency room liaison teams, clinical evaluation tools and training activities. The Health and Social Services Ministry prioritised implementation of emergency room liaison teams in a context where emergency departments were being overwhelmed by population demand [36]. The emergency room liaison teams promoted rapid assessment and referral to appropriate services for untreated individuals with substance-use disorders, as well as increased service coordination [68]. According to a recent study, substance-use disorder clients identified by emergency room liaison teams were 30 times more likely than others to enter a therapeutic programme [69]. Screening and brief intervention by emergency room nurses also lowered emergency readmission rates [70] and helped alleviate overcrowding in the emergency room [71]. Moreover, Desy et al. [70] found reduced rates of alcohol use among 70% of substance-use disorder clients identified by emergency room nurses, as opposed to 20% of those who received usual care.

Standardised clinical evaluation tools improve the capacity to identify individuals with substance-use disorders [72], and also help increase referrals, especially from primary care to specialised services. Participants reported that the clinical evaluation tool used in local community service centres was effective. However, the complexity of the instrument and time required for completion tended to deter primary-care clinicians from using it. They recommended development of a simplified clinical tool similar to the brief version of the Global Appraisal of Individual Needs, which was produced as a screening measure for adolescents [72].

Concerning training activities, the availability of a good training infrastructure during the implementation of reforms reduces staff turnover [73]. Primary-care providers who received basic training on substance-use disorder screening and intervention were more capable of conducting brief interventions and making appropriate referrals. A number of countries have implemented the Screening, Brief Intervention, and Referral to Treatment protocol in primary care [74,75]. A recent literature review demonstrated the efficacy of Screening, Brief Intervention, and Referral to Treatment for reducing alcohol abuse, especially among middle-aged individuals (20–64 years) [75]. There are also reports of promising results from use of the Screening, Brief Intervention, and Referral to Treatment among drug abusers [76,77]. Interagency training conducted with an interdisciplinary approach also encouraged exchanges, communication and support among clinicians. Cross-training is one of the most effective types of training for knowledge translation around various substance-use disorder issues and concurrent disorders (e.g. mental health disorders) and around intervention methods [78]. One study found that training on co-occurring disorders improved job satisfaction and morale among professionals working with this clientele [79]. Cross-training has also been found useful in supporting improved service integration [78] and in reducing differences among the visions and practices of professionals in diverse organisations.

Clinical integration is central to the service integration process, but must be supported by administrative integration strategies [80]. In Quebec, the latter have been more adequately implemented in primary care, reinforcing substance-use disorder services. The fact that most individuals with, or at risk for, substance-use disorders use primary-care services, especially those of general practitioners, provides the justification for the focus on primary care [75]; only a minority seek help from specialised services [81]. Moreover, primary-care services are considered less stigmatising and take a more comprehensive approach, as they manage substance-use disorders along with physical problems or mental health disorders [82]. Finally, robust primary-care systems are thought to achieve better organisational and population health outcomes [29,61,83] and to reduce overall healthcare costs [31,61].

The first two substance-use disorder primary-care models identified in this study involved the sharing of expertise by a substance-use disorder consultant with other clinicians and managers in the organisation. Similar to an existing model in California [61], services were not offered directly to substance-use disorder clients by the consultant. Instead, the approach adopted resembled the consultation liaison model that was introduced to the Quebec mental healthcare system through the practice of shared-care, in which designated psychiatrists provide assistance to general practitioners [84]. The consultation liaison model was shown in one meta-analysis to improve the capacity of general practitioners to prescribe pharmacological therapy and to treat individuals with mental health disorders more adequately [85]. Yet further research would be needed to demonstrate the effectiveness of the consultation liaison model for the treatment of substance-use disorders. The second model built on the previous model, adding caseworkers appointed from each programme (e.g. programmes for mental health disorders) who were assigned to the substance-use disorder consultant for support. This approach was considered one way of keeping substance-use disorder issues alive within each team. This model also holds promise since caseworkers maintain direct contact with clients, while acting as substance-use disorder resource clinicians and change agents on their respective teams. This second model was found difficult to implement, however, as some teams were less mobilised than others regarding work with substance-use disorder clients due to lack of interest or prejudice. As well, substance-use disorder was generally given secondary priority to the targeted programme, for example services for mental health disorders. Finally, given the prevalence of clients with co-occurring disorders, the recommended strategy was to integrate a key substance-use disorder worker into mental health disorder teams (Model 3) or into primary-care teams in territories with a high prevalence of substance-use disorder clients [53]. Model 3 has been implemented primarily in rural sites or under conditions of scarce resources.

Findings revealed that information and monitoring tools were not adequately implemented as yet. The Quebec information and monitoring system for managing administrative and clinical information represents an important asset. However, this system was not routinely used by clinicians and could be much improved in terms of the addition of follow-up data. The implementation of a strong health information technology infrastructure is recognised in the literature as a key to effective care coordination [57].

Overall, the integration strategies implemented in Quebec did not seem to eliminate gaps in follow-up or in continuity of care among individuals with substance-use disorders. Nor did they increase primary-care service delivery or improve service integration within the substance-use disorder or the mental health disorder programmes. Some countries have introduced case management as a remedy for the lack of continuity of care among individuals with substance-use disorders, especially those with chronic and complex profiles [86]. Case management helps substance users acquire skills needed for daily living, promotes their integration into society and prevents hospitalisation. The US Substance Abuse and Mental Health Services Administration [87] proposes four models of case management adapted for substance-use disorders: assertive community treatment, the broker model, the strengths-based approach and the clinical management model. Meta-analyses found that case management was superior to conventional treatment as a tool for successful liaison among services [14,88].

The literature suggests inclusion of a substance-use disorder expert in mental health disorder services, as well as training on blended services as a way of promoting service integration for co-occurring disorders [89]. Shared care, which brings together general practitioners and specialised care providers, could also be implemented in the substance-use disorder system, as has been done successfully with mental health disorders [90]. Also, demonstration projects have established the feasibility of several integrated substance-use disorder treatment models [63]. The chronic care model [91], for example, combines medical and psychosocial approaches for assessing and treating individuals with substance-use disorders. This model has been implemented in several countries for managing diabetes, heart disease, cancer and other chronic diseases, but also in the management of mental health disorders, specifically depression [92]. The tiered model, inspired by the stepped-care model used in the United Kingdom for treating depression, has been adopted by some Canadian provinces (Alberta, British Columbia and New Brunswick) to support the coordination of mental health disorder and substance-use disorder services [93]. In the case of stepped-care, interventions are performed hierarchically according to the severity level of the substance-use disorder or risk level to the individual. The need for collaborative strategies thus increases in relation to the severity and complexity of the substance-use disorder profile [93].

## Limitations

This study had a number of limitations. First, the results may not be generalisable to other areas of Quebec or to other countries, as they reflect the characteristics of local networks and areas. Second, study participants may have over- or under-estimated the actual level of integration in their local service networks. In order to neutralise participant bias, we validated their comments with relevant documents from organisations and local service networks. Finally, there were very few diverging viewpoints among participants, but rather the information gathered was complementary or convergent.

## Conclusion

This is the first study to present the Quebec Substance-use Disorder Programme, and the key strategies used in promoting successful network integration. There are few available studies related to substance-use disorder systems that provide a macro-level perspective on integrated care. Our study shows that the implementation of few integration strategies is insufficient to bring about successful integration in complex service networks In Quebec, a major investment was made in terms of the screening and assessment of individuals with substance-use disorders by implementing standardised clinical evaluation tools and training initiatives. Primary-care models, emergency room liaison teams and information systems were identified as the integration strategies most likely to increase integration and to provide better access to Quebec substance-use disorder services. Therefore, it is important to implement additional clinical projects and planning initiatives in order to better understand supply and demand for substance-use disorder services and to facilitate the development of a continuum of care that meets the needs of various substance-use disorder clients. Promoting the consolidation of substance-use disorder services in primary care and creating links with specialised services through a greater standardisation of referral mechanisms is crucial to ensure continuous follow-up of substance-use disorder clients. Finally, strategies need to be systematically employed for better integration of substance-use disorder services, at both clinical (e.g. case management) and administrative (e.g. service contract) levels.

## Competing Interests

The authors declare that they have no competing interests.

## References

[1] Hasin DS, Stinson FS, Ogburn E, Grant BF (2007). Prevalence, correlates, disability, and comorbidity of DSM-IV
alcohol abuse and dependence in the United States: results from the National
Epidemiologic Survey on Alcohol and Related Conditions. Archives of General Psychiatry.

[2] Schulden JD, Thomas YF, Compton WM (2009). Substance abuse in the United States: findings from recent
epidemiologic studies. Current Psychiatry Reports.

[3] Pearson C, Janz T, Ali J (2013). Mental and substance use disorders in Canada. Health at a glance.
Statistics Canada Catalogue.

[4] Statistics Canada. Canadian Community Health Survey (CCHS) (2002).

[5] Castel S, Rush B, Urbanoski K, Toneatto T. (2006). Overlap of clusters of psychiatric symptoms among clients of a
comprehensive addiction treatment service. Psychology of Addictive Behaviors.

[6] Compton WM, Thomas YF, Stinson FS, Grant BF (2007). Prevalence, correlates, disability, and comorbidity of DSM-IV
drug abuse and dependence in the United States: results from the national
epidemiologic survey on alcohol and related conditions. Archives of General Psychiatry.

[7] Colpaert K, Vanderplasschen W, van Hall G, Broekaert E, Schuyten G (2008). Dual substance abusers seeking treatment: demographic,
substance-related, and treatment utilization characteristics. Journal of Drug Issues.

[8] Kessler RC (2004). The epidemiology of dual diagnosis. Biological Psychiatry.

[9] Isaacs S, Jellinek P, Garcel JM, Hunt KA, Bunch W (2013). New York state health foundation: integrating mental health and
substance abuse care. Health Affairs.

[10] Copello A, Walsh K, Graham H, Tobin D, Griffith E, Day E (2013). A consultation-liaison service on integrated treatment: a program
description. Journal of Dual Diagnosis.

[11] Wu LT, Swartz MS, Wu Z, Mannelli P, Yang C, Blazer DG (2012). Alcohol and drug use disorders among adults in emergency
department settings in the United States. Annals of Emergency Medicine.

[12] Mojtabai R (2005). Use of specialty substance abuse and mental health services in
adults with substance use disorders in the community. Drug & Alcohol Dependence.

[13] Cleary M, Hunt GE, Matheson S, Walter G (2009). Views of Australian mental health stakeholders on clients’
problematic drug and alcohol use. Drug and Alcohol Review.

[14] Rapp RC, Otto AL, Lane DT, Redko C, McGatha S, Carlson RG (2008). Improving linkage with substance abuse treatment using brief case
management and motivational interviewing. Drug and Alcohol Dependence.

[15] Riley ED, Wu AW, Junge B, Marx M, Strathdee SA, Vlahov D (2002). Health services utilization by injection drug users participating
in a needle exchange program. American Journal of Drug & Alcohol Abuse.

[16] Darke S, Ross J, Teesson M, Lynskey M (2003). Health service utilization and benzodiazepine use among heroin
users: findings from the Australian treatment outcome study
(ATOS). Addiction.

[17] Lee SYD, Morrissey JP, Thomas KC, Carter WC, Ellis AR (2006). Assessing the service linkages of substance abuse agencies with
mental health and primary care organizations. American Journal of Drug and Alcohol Abuse.

[18] Hu HM, Kline A, Huang FY, Ziedonis DM (2006). Detection of co-occurring mental illness among adult patients in
the New Jersey substance abuse treatment system. American Journal of Public Health.

[19] Vanderplasschen W, De Bourdeaudhuij I, Van Oost P (2002). Co-ordination and continuity of care in substance abuse
treatment. An evaluation study in Belgium. European Addiction Research.

[20] Drake RE, Mueser KT, Brunette MF, McHugo GJ (2004). A review of treatments for people with severe mental illnesses
and co-occurring substance use disorders. Psychiatric Rehabilitation Journal.

[21] Mueser KT, Torrey WC, Lynde D, Singer P, Drake RE (2003). Implementing evidence-based practices for people with severe
mental illness. Behavioral Modification.

[22] Drake RE, Wallach MA (1988). Mental patients’ attitudes toward hospitalization: a
neglected aspect of hospital tenure. American Journal of Psychiatry.

[23] Brooks AJ, Penn PE (2003). Comparing treatments for dual diagnosis: twelve-step and
self-management and recovery training. American Journal of Drug and Alcohol Abuse.

[24] Essock SM, Mueser KT, Drake RE, Covell NH, McHugo GJ, Frisman LK (2006). Comparison of ACT and standard case management for delivering
integrated treatment for co-occurring disorders. Psychiatric Services.

[25] Donald M, Dower J, Kavanagh D (2005). Integrated versus non-integrated management and care for clients
with co-occurring mental health and substance use disorders: a qualitative
systematic review of randomised controlled trials. Social Science & Medicine.

[26] Glasner-Edwards S, Tate SR, McQuaid JR, Cummins K, Granholm E, Brown SA (2007). Mechanisms of action in integrated cognitive-behavioral treatment
versus twelve-step facilitation for substance-dependent adults with comorbid
major depression. Journal of Studies on Alcohol and Drugs.

[27] Ball SA (2007). Comparing individual therapies for personality disordered opioid
dependent patients. Journal of Personality Disorders.

[28] Gryczynski J, Mitchell SG, Peterson TR, Gonzales A, Moseley A, Schwartz RP (2011). The relationship between services delivered and substance use
outcomes in New Mexico’s Screening, Brief Intervention, Referral and
Treatment (SBIRT) Initiative. Drug & Alcohol Dependence.

[29] Madras BK, Compton WM, Avula D, Stegbauer T, Stein JB, Clark HW (2009). Screening, brief interventions, referral to treatment (SBIRT) for
illicit drug and alcohol use at multiple healthcare sites: comparison at
intake and 6 months later. Drug & Alcohol Dependence.

[30] Parthasarathy S, Mertens J, Moore C, Weisner C (2003). Utilization and cost impact of integrating substance abuse
treatment and primary care. Medical Care.

[31] Babor TF, McRee BG, Kassebaum PA, Grimaldi PL, Ahmed K, Bray J (2007). Screening, brief intervention, and referral to treatment (SBIRT):
toward a public health approach to the management of substance
abuse. Substance Abuse.

[32] Fleury MJ (2006). Integrated service networks: the Quebec case. Health Services Management Research.

[33] Shortell SM, Gillies RR, Anderson DA (1994). The new world of managed care: creating organized delivery
systems. Health Affairs.

[34] Quebec Association of Addiction Rehabilitation Centers (2014). Le privilège du pouvoir. Portrait d’un réseau
public spécialisé aux pratiques éprouvées. [The
privilege of power. A portrait of a specialised public sector with proven
practices.].

[35] Fleury MJ, Grenier G (2012). État de situation sur la santé mentale au Québec
etréponse du système de santé et de services sociaux. [The
Quebec mental health system: synthesis of knowledge about the system
performance.].

[36] Quebec Ministry of Health Social Services (2007). Unis dans l’action: Offre de service 2007–2012
Programme-service Dépendances: Orientations relatives aux standards
d’accès, de continuité, de qualité,
d’efficacité et d’efficience. [United in the action:
services offered 2007–2012 Addiction program: orientations relative to
standards of access, continuity, quality, efficacy and
efficiency.].

[37] Morgan D, Krueger RA (1997). The focus group kit.

[38] Titscher S, Wodak R, Meyer M, Vetter E (2000). Methods of text and discourse analysis.

[39] Agency of Health Social Services of Estrie (AHSS) (2009). Au coeur du PSI: la personne et ses proches. [At the heart of the
Individual service plan: the individual and his relatives.].

[40] Intagliata J (1982). Improving the quality of community care for the chronically
mentally disabled: the role of case management. Schizophrenia Bulletin.

[41] Drake RE, Mueser KT, Torrey WC, Miller AL, Lehman AF, Bond GR (2000). Evidence-based treatment of schizophrenia. Current psychiatry Reports.

[42] Nelson G, Aubry T, Lafrance A (2007). A review of the literature on the effectiveness of housing and
support, assertive community treatment, and intensive case management
interventions for persons with mental illness who have been
homeless. American Journal of Orthopsychiatry.

[43] Tremblay J, Rouillard P, Sirois M (2001). Dépistage/évaluation du besoin d’aide –
Alcool/Drogues/Jeu. [Screening/Assessment of the need of
help-Alcohol/Drugs/Gambling.].

[44] McLellan AT, Luborsky L, Woody GE, O’Brien CP (1980). An improved diagnostic evaluation instrument for substance abuse
patients: the addiction severity index. Journal of Nervous and Mental Disease.

[45] Dennis ML, White M, Titus J, Unsicker J (2008). Global appraisal of individual needs (GAIN): administration guide
for the GAIN and related measures (Version 5).

[46] Health Canada (2002). Best practices-concurrent mental health and substance use
disorders.

[47] Simmonds M (2003). Cross-training for collaborative systems prevention, treatment
and care. http://www.treatment.org/topics/infectious.html.

[48] Drake RE, Mueser KT (1996). Dual diagnosis of major mental illness as substance abuse (Vol.
2): recent research and clinical implications.

[49] Riper H, Andersson G, Hunter SB, de Wit J, Berking M, Cuijpers P (2014). Treatment of comorbid alcohol use disorders and depression with
cognitive-behavioural therapy and motivational interviewing: a
meta-analysis. Addiction.

[50] Quebec Ministry of Health Social Services (2007). Alcochoix+. Implanter et promouvoir. [Alcochoice +.
Implementation and promotion.].

[51] Quebec Association of Addiction Rehabilitation Centers (2014). Mémoire de l’Association des centres de
réadaptation en dépendance du Québec sur le projet de loi No
10, Loi modifiant l’organisation et la gouvernance du réseau de
la santé et des services sociaux notamment par l’abolition des
agences régionale, déposé à la Commission de la
santé et des services sociaux le 23 octobre 2014 [Brief presented by
the Association of Quebec Addiction Rehabilitation Centres on the proposed
Bill 10 with respect to modification in the organisation and governance of
the health and social service network, notably by abolishing the regional
Agencies].

[52] Roberts B (2012). Interprofessional relationships in dual diagnosis discourse in an
Australian State: are we respecting each other yet?. Mental Health and Substance Use: Dual Diagnosis.

[53] Gurewich D, Prottas J, Sirkin JT (2014). Managing care for patients with substance abuse disorders at
community health centers. Journal of Substance Abuse Treatment.

[54] Kessler R, Stafford D, Messier R (2009). The problem of integrating behavioral health in the medical home
and the questions it leads to. Journal of Clinical Psychology In Medical Settings.

[55] Cherry AL, Zarrow A, Zarrow H, L’abate L (2011). Mixing oil and water: Developing integrated treatment for people
with the co-occurring disorders of mental illness and
addiction. Mental illness – evaluation, treatments and implication.

[56] Hintz T, Mann K (2006). Co-occurring disorders: policy and practice in
Germany. American Journal of Addiction.

[57] Mi-Case (2012). Addressing substance abuse treatment in the United States –
Insights from the UK.

[58] Rush BR, Nadeau L, Cooper BD (2011). Integrated services and system planning debate. Responding in mental health-substance use.

[59] Chalk M, Dilonardo J, Gelber Rinaldo S (2011). Purchasing integrated services for substance use conditions in
health care settings: an issue briefs of lessons learned and challenges
ahead. Forum of integration.

[60] Sacks S, Chaple M, Sirikantraporn J, Sacks JY, Knickman J, Martinez J (2013). Improving the capability to provide integrated mental health and
substance abuse services in a state system of outpatient
care. Journal of Substance Abuse Treatment.

[61] Pawda H, Urada D, Antonini VP, Ober A, Crèvecoeur-MacPhail DA, Rawson A (2012). Integrating substance use disorder services with primary care:
the experience of California. Journal of Psychoactive Drugs.

[62] van Boekel LC, Brouwers EP, van Weeghel J, Garretsen HF (2014). Healthcare professionals’ regard towards working with
patients with substance use disorders: comparison of primary care, general
psychiatry and specialist addiction services. Drug and Alcohol Dependence.

[63] Pating DR, Miller MM, Goplerud E, Martin J, Ziedonis DM (2012). New systems of care for substance use disorders: treatment,
finance, and technology under health care reform. Psychiatric Clinics of North America.

[64] Dill A, Rochefort DA (1989). Coordination, continuity and centralized control: a policy
perspective on services strategies to the chronically mentally
ill. Journal of Social Issues.

[65] Provan KG, Sebastian JG, Milward HB (1996). Interorganizational cooperation in community mental health: a
resource-based explanation of referrals and case
coordination. Medical Care Research and Review.

[66] Torrey WC, Tepper M, Greenwold J (2011). Implementing integrated services for adults with co-occurring
substance use disorders and psychiatric illnesses: a research
review. Journal of Dual Diagnosis.

[67] Marshall KL, Deane FP (2004). General practitioners’ detection and management of patients
with a dual diagnosis: Implications for education and
training. Drug and Alcohol Review.

[68] Sainte-Marie T, Peyré A, Paille F, Fleury B, Landry M, Brochu S, Pateneaude C (2012). Évolution des équipes de liaison et de soins en
addictologie (ELSA) en France 1999–2010. Évolution des
équipes de liaison et de soins en addictologie (ELSA) en France
1999–2010. [Evolution of liaison and addiction care teams in France
from 1999 to 2010.].

[69] D’Onofrio G, Degutis LC (2010). Integrating Project ASSERT: a screening, intervention, and
referral to treatment program for unhealthy alcohol and drug use into an
urban emergency department. Academic Emergency Medicine.

[70] Desy PM, Howard PK, Perhats C, Li S (2010). Alcohol screening, brief intervention, and referral to treatment
conducted by emergency nurses: an impact evaluation. Journal of Emergency Nursing.

[71] Vermette G (2008). Guide d’implantation. Équipe de liaison
spécialisée en dépendances à l’urgence.
[Implementation guideline. Liaison teams specialized in addiction to the
emergency-rooms.].

[72] McDonell MG, Comtois KA, Voss WD, Morgan AH, Ries RK (2009). Global appraisal of individual needs short screener (GSS):
psychometric properties and performance as a screening measure in
adolescents. American Journal of Drug & Alcohol Abuse.

[73] Woltmann EM, Whitley R (2007). The role of staffing stability in the implementation of
integrated dual disorders treatment: an exploratory study. Journal of Mental Health.

[74] Heather N, Dallolio E, Hutchings D, Kaner E, White M (2004). Implementing routine screening and brief alcohol intervention in
primary health care: a Delphi survey of expert opinion. Journal of Substance Use.

[75] O’Donnell A, Anderson P, Newbury-Birch D, Schulte B, Schmidt C, Reimer J (2014). The impact of brief alcohol interventions in primary healthcare:
a systematic review of reviews. Alcohol and Alcoholism.

[76] Bernstein J, Bernstein E, Tassiopoulos K, Heeren T, Levenson S, Hingson R (2005). Brief motivational intervention at a clinic visit reduces cocaine
and heroin use. Drug & Alcohol Dependence.

[77] Baker A, Lee NK, Claire M, Lewin TJ, Grant T, Pohlman S (2005). Brief cognitive behavioural interventions for regular amphetamine
users: a step in the right direction. Addiction.

[78] Perreault M, Wiethaueper D, Perreault N, Bonin JP, Brown TG, Brunaud H (2009). Meilleures pratiques et formation dans le contexte du continuum
des services en santé mentale et en toxicomanie: le programme de
formation croisée du sud-ouest de Montréal. [Cross training
program in Montreal’s south west: best practices and training in a
context of continuum of services in mental health care and addiction
treatment]. Santé mentale au Québec.

[79] Hunter SB, Watkins KE, Wenzel S, Gilmore J, Sheehe J, Griffin B (2005). Training substance abuse treatment staff to care for co-occurring
disorders. Journal of Substance Abuse Treatment.

[80] Craven MA, Bland R (2006). Better practices in collaborative mental health care: an analysis
of the evidence base. Canadian Journal of Psychiatry.

[81] Urbanoski KA, Rush BR, Wild TC, Bassani DG, Castel S (2007). Use of mental health care services by Canadians with co-occurring
substance dependence and mental disorders. Psychiatric Services.

[82] Fleury MJ, Imboua A, Aube D, Farand L, Lambert Y (2012). General practitioners’ management of mental disorders: a
rewarding practice with considerable obstacles. BMC Family Practice.

[83] Starfield B, Lemke KW, Bernhardt T, Foldes SS, Forrest CB, Weiner JP (2003). Comorbidity: implications for the importance of primary care in
‘case’ management. Annals of Family Medicine.

[84] Quebec Ministry of Health Social Services (2005). Plan d’action en santé mentale 2005–2010 –
La force des liens. [Mental Health Action Plan 2005–2010 – the
strength of the links.].

[85] Gillies D, Buykx P, Parker AG, Hetrick SE (2015). Consultation liaison in primary care for people with mental
disorders. Cochrane Database System Review.

[86] Vanderplasschen W, Mostien B, Franssen A, Lievens K, De Maeyer J, Broekaert E (2007). Dealing with multiple and frequent service utilisation in
substance abuse treatment: experiences with coordination of care in
residential substance abuse agencies in the region of Ghent,
Belgium. Therapeutic Communities.

[87] Substance Abuse Mental Health Administration (SAMHSA) (2012). Comprehensive case management for substance abuse
treatment.

[88] Hesse M, Vanderplasschen W, Rapp RC, Broekaert E, Fridell M (2007). Case management for persons with substance use
disorders. Cochrane Database of Systematic Reviews.

[89] Brousselle A, Lamothe L, Sylvain C, Foro A, Perreault M (2010). Integrating services for patients with mental and substance use
disorders: what matters?. Health Care Management Review.

[90] Kates N, McPherson-Doe C, George L (2011). Integrating mental health services within primary care settings:
the Hamilton Family Health Team. Journal of Ambulatory Care Management.

[91] Saitz R, Cheng DM, Winter M, Kim TW, Meli SM, Allensworth-Davies D (2013). Chronic care management for dependence on alcohol and other
drugs. The AHEAD randomized trial. Journal of the American Medical Association.

[92] Blakely TJ, Dziadosz GM (2007). Creating an agency integrated treatment program for co-occurring
disorders. American Journal of Psychiatric Rehabilitation.

[93] Canadian Centre on Substance Abuse (2014). Collaboration for addiction and mental health care: best
advice.

